# Clinicopathological Characteristics of Primary Pulmonary Hodgkin Lymphoma (PPHL): Two Institutional Experiences with Comprehensive Literature Review of 115 PPHL Cases

**DOI:** 10.3390/jcm12010126

**Published:** 2022-12-23

**Authors:** Hera Jung, Hyun-Soo Kim, Joungho Han, Young Hyeh Ko, Yoo-Duk Choi, Taebum Lee

**Affiliations:** 1 Department of Pathology, CHA Ilsan Medical Center, CHA University School of Medicine, Goyang 10414, Republic of Korea; 2 Department of Pathology and Translational Genomics, Samsung Medical Center, Sungkyunkwan University School of Medicine, Seoul 06351, Republic of Korea; 3 Department of Pathology, Korea University Guro Hospital, Korea University College of Medicine, Seoul 10408, Republic of Korea; 4 Department of Pathology, Chonnam National University Hospital, Chonnam National University Medical School, Gwangju 61469, Republic of Korea

**Keywords:** lung, Hodgkin lymphoma, differential diagnosis, literature review

## Abstract

Primary pulmonary Hodgkin lymphoma (PPHL) is an extremely rare condition. Its clinicopathological characteristics remain unclear because of the limited number of patients with PPHL. The aim of this study was to comprehensively analyze the clinicopathological characteristics of PPHL. We reviewed the electronic medical records and pathology slides of our 10 PPHL patients. The female-to-male ratio was 6:4, and the mean age was 41 years. Although three patients had no symptoms, seven had localized or generalized symptoms, including cough, sputum, chest discomfort/pain, and weight loss. Some cases had not been diagnosed as PPHL in the initial needle biopsy. Four patients underwent surgical resection. With chemotherapy, eight patients achieved complete remission. We also conducted a thorough literature review on 105 previously reported PPHL cases. Among a total of 115 PPHL cases, the most common subtype was nodular sclerosis (37.4%). More than half of the cases (55%) were clinically suspected as infectious pneumonia. Of 61 patients whose biopsies were available, 27 (44.3%) were diagnosed correctly as Hodgkin lymphoma, whereas the misdiagnoses included tuberculosis, Langerhans cell histiocytosis, solitary fibrous tumor, and adenocarcinoma. We demonstrated that PPHL represents a diagnostic challenge on small biopsies. Recognizing that this rare tumor can mimic infectious and inflammatory diseases as well as malignancies is important because the accurate diagnosis of PPHL is essential for adequate clinical management.

## 1. Introduction

Hodgkin lymphoma (HL) was initially described in 1832 by Thomas Hodgkin [1]. HL is now divided into classic and nodular lymphocyte-predominant types based on histological features and immunophenotypes [2]. Epidemiologically, approximately 90% of HLs are classic type. Classic HL has four subtypes: nodular sclerosis, lymphocyte-rich, mixed cellularity, and lymphocyte depleted [2]. Classic HL is characterized by a Reed–Sternberg (RS) cell which is a large atypical cell possessing abundant basophilic cytoplasm and bilobed or polylobed nucleus [3]. While having no classic Reed–Sternberg cells, nodular lymphocyte-predominant HL is composed of lymphocytic and/or histiocytic (L&H) type of cell (“popcorn” cell) [3].

Most cases of HL arise in lymph nodes including the cervical, mediastinal, axillary, and para-aortic [2]. Meanwhile, primary extranodal involvement of HL is quite uncommon and especially, primary pulmonary HL (PPHL) is extremely rare and has been known as less than 1% of all lymphomas [4,5]. When HL restrictively involves pulmonary parenchyma with no extrapulmonary spread at the time of diagnosis can fulfill the diagnostic criteria of PPHL [6]. However, due to its low prevalence, clinicopathological features of PPHL have been unclear. Additionally, histological diagnosis of PPHL is often challenging in a small biopsy specimen, including fine needle aspiration cytology [7].

For about 25 years, a total of 10 cases of PPHL were diagnosed at Samsung Medical Center (Seoul, Republic of Korea) and Chonnam National University Hospital (Gwangju, Republic of Korea) by pathological confirmation. This study presents clinical, epidemiological, radiological, and histological features of 10 PPHL cases diagnosed in two institutions. We also conduct a comprehensive literature review with an integrated analysis of the clinicopathological features of 115 PPHL cases (including our 10 cases). Some of the cases were misdiagnosed in precedent needle biopsies. By its inflammatory background, PPHL can mimic other infective diseases, such as tuberculosis and inflammatory lesions. There have also been misdiagnosed cases of mesenchymal tumors, histiocytic neoplasms, and epithelial malignancies. Especially, considering the difficulty of diagnosis in small samples, we compare the histological and immunohistochemical characteristics of the diseases that were misdiagnosed through the literature review.

## 2. Materials and Methods

### 2.1. Case Selection and Clinicopathological Data Collection

Between January 1995 and February 2019, the pathology databases of the Samsung Medical Center and the Chonnam National University Hospital were searched for pulmonary biopsies and resections with keywords including ‘Hodgkin disease’ or ‘Hodgkin lymphoma’. Two board-certified pathologists specialized in hematopathology reviewed all available hematoxylin and eosin-stained slides. The diagnoses of PPHL were established according to the following diagnostic criteria [6,8]: (1) compatible histological features of HL; (2) restriction of the disease to the lungs, with or without minimal hilar lymph node involvement; and (3) adequate clinical and pathological exclusion of the disease at a distant site. We excluded secondary pulmonary involvement by primary nodal or mediastinal HL. During a 25-year study period, 10 cases of PPHL were found. The following clinical information was collected from electronic medical records: demographic features, clinical symptoms, imaging findings, treatment history, and outcomes. The most representative slide for each case was chosen for immunohistochemical staining and Epstein–Barr virus (EBV)-encoded RNA in situ hybridization (EBER-ISH).

### 2.2. Immunohistochemical Staining

We performed immunostaining using the Ventana Benchmark XT automated staining system (Ventana Medical Systems, Oro Valley, AZ, USA). Formalin-fixed, paraffin-embedded (FFPE) tissue sections were deparaffinized and rehydrated with a xylene and alcohol solution. After antigen retrieval, endogenous peroxidases were quenched with hydrogen peroxide. The sections were incubated with primary antibodies against CD 30 (dilution 1:30, Dako, Glostrup, Denmark), CD15 (dilution 1:100, Dako), CD20 (dilution 1:200, Dako), CD3 (dilution 1:200, Dako), CD4 (dilution 1:100, Dako), and paired box 5 (PAX5; prediluted, Novocastra, Leica Biosystems, Newcastle upon Tyne, UK). After chromogenic visualization using 3,3′-diaminobenzidine, the sections were counterstained with hematoxylin, and then embedded in a mounting solution. Appropriate controls were stained concurrently. Positive controls were tonsils for CD30, CD15, CD20, CD3, CD4 and PAX5. Negative controls were prepared by substituting non-immune serum for primary antibodies, which resulted in undetectable staining.

### 2.3. EBER-ISH

The presence of EBV in the tumor cells was confirmed by the expression of EBER1, the most abundant viral product in latently infected cells. We performed EBER-ISH using the Bond III automated staining system (Leica Biosystems, Wetzlar, Germany). FFPE tissue sections were deparaffinized with xylene, pretreated with proteinase K for 20 min, and incubated with fluorescein isothiocyanate (FITC)-conjugated EBER oligonucleotide probes (Novocastra) at 55 °C for 2 h. The sections were rinsed in water and incubated with horseradish peroxidase-conjugated anti-FITC antibody for 15 min before adding the chromogen to produce an alcohol-insoluble dark nuclear staining in the EBV-positive cells. We used EBV-negative lymphoid tissues processed using the hybridization mixture without EBER oligonucleotides as negative controls.

### 2.4. Comprehensive Literature Review

The Medline database was thoroughly searched using PubMed retrieval service. The keywords used were “primary pulmonary Hodgkin lymphoma.” 15 cases with features suspicious for or suggestive of secondary pulmonary involvement were eliminated. We collected the following clinicopathological information from a total of 115 PPHL cases including ours and 105 previously published cases: sex, age, imaging findings, regional lymph node involvement, clinical impression, and subtype.

### 2.5. Statistical Analysis

Fisher’s exact test was used to examine whether there are significant differences in clinicopathological characteristics between ours and previous cases. All statistical analyses were performed using IBM SPSS Statistics for Windows, version 23.0 (IBM Corp., Armonk, NY, USA). Statistical significance was defined as *p* < 0.05.

## 3. Results

### 3.1. Clinical Features

**Figure 1:**
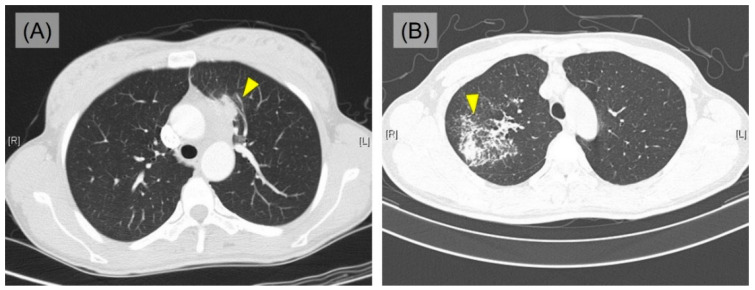
Imaging findings of PPHL. (**A**) Single mass (yellow arrowhead; case 4). (**B**) Consolidation (yellow arrowhead; case 9).

The clinical data of the 10 PPHL cases are summarized in Table 1. Detailed clinicopathological characteristics are listed in Supplementary Table S1. The median observation period was 4.5 years (range, from 1 month to 21 years). The median age of patients at diagnosis was 41 years (range, from 27 to 72 years), and female predilection was identified (female: male = 6:4). Three patients had a history of smoking (range, 1 to 40 pack years). Seven patients complained of localized and/or generalized symptoms; cough, sputum, chest discomfort/pain, and weight loss, but the rest of 3 patients were asymptomatic. In the initial chest computed tomography (CT), 8 patients presented pulmonary mass (size range, from 4.1 to 10.5 cm) accompanying enlarged mediastinal lymph nodes, which highly indicated primary lung cancer (Figure 1A). Two patients showed consolidation, which suggests the possibility of tuberculosis or nontuberculous mycobacterial infection (Figure 1B) and pneumonia. Most of the masses and consolidative lesions were located in upper portion of the lung (5 in left upper lobe and 4 in right upper lobe), except one which was identified in left lower lobe. The bronchoscope examination was performed on 8 patients and most of them had no specific findings except 2 cases showing equivocal narrowing and a suspicious mucosal nodular infiltrating lesion, each.

### 3.2. Histological and Immunohistochemical Features

**Figure 2:**
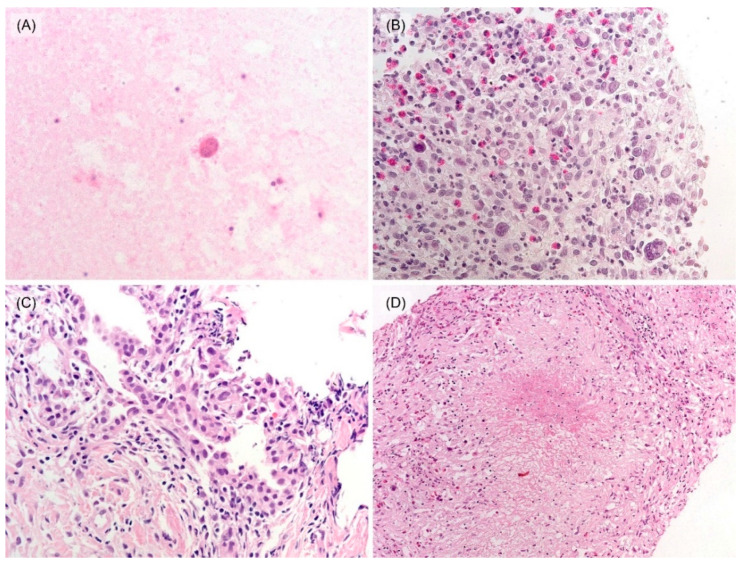
Histologic features of the biopsy specimen. (**A**) Aspiration cytology (case 2): a single RS cell is identified in the bloody background (400×). (**B**) Needle biopsy (case 5): a few RS cells are admixed with inflammatory cells, including eosinophils and histiocytes (400×). (**C**) Needle biopsy (case 4): activated pneumocytes in PPHL can mimic lung adenocarcinoma (400×). (**D**) Needle biopsy (case 6): histiocytic aggregates and necrosis, mimicking tuberculosis (200×).

**Figure 3:**
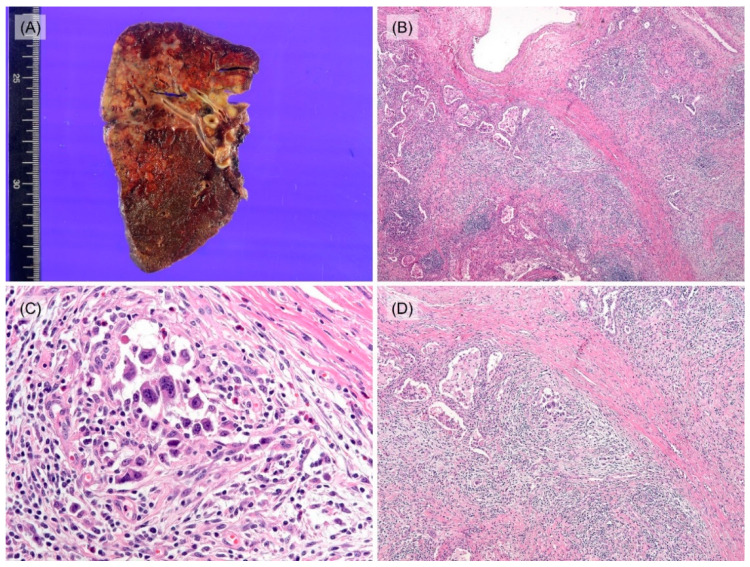
Gross and histologic features of resected specimen (case 9). (**A**) Gross finding of right upper lobectomy specimen shows multiple yellowish nodules with septation. (**B**) Some reactive epithelial cells in the inflammatory background are showing moderate atypia; thus, it is difficult to distinguish between epithelial malignancy and PPHL (40×). (**C**) Hodgkin cells in the inflammatory background (400×). (**D**) Activated pneumocytes in peripheral area (100×).

Sputum smear and/or bronchial washing cytology were performed in seven patients and all results were negative for malignant cells. Four patients underwent percutaneous needle aspiration, and nine patients underwent needle biopsy. Most of the results of aspiration cytology were insufficient for diagnosis except for one suspicious case with a single RS-like cell (Figure 2A). Six patients were diagnosed with HL by initial needle biopsy, showing a few RS cells mixed with many inflammatory cells, including lymphocytes, eosinophils, and histiocytes (Figure 2B). Among these, four cases were classified by subtypes: two mixed cellularity, one nodular sclerosis, and one lymphocyte-rich. Additionally, the others were insufficient for classifying subtypes, due to the small amount of tissue. One case was diagnosed as ‘suggestive of HL’ histologically based on many eosinophils and histiocytes without the presence of typical RS cells and atypical tumor cells showed positivity for CD30 and PAX5 while CD3, CD20, and EBER-ISH were negative (Supplementary Table S2). One of the cases showed atypical pneumocytes suggesting the possibility of adenocarcinoma (Figure 2C) in an initial biopsy, thus she received neoadjuvant chemotherapy (TC; docetaxel, and cisplatin, #1) before surgical resection. Two patients, who were suspected of tuberculosis in needle biopsy (Figure 2D) and chest CT, were treated with anti-tuberculosis medication for 3 months, however, the extent of lesions increased in follow-up chest CT. The patient who was suspected of pneumonia in radiological findings was administered for antibiotic treatment, but the lesion was aggravated. After needle biopsy, he was finally diagnosed as mixed cellularity PPHL. Among four patients who underwent surgical resection, intraoperative frozen biopsy was conducted for three of them, and results were ‘No evidence of malignancy and necrofibrotic nodule due to previous neoadjuvant chemotherapy’, ‘Malignant tumor’ and ‘Atypical cells present in the inflammatory and fibrotic background’, each. These three cases were finally diagnosed as PPHL in the permanent section. Grossly resected lung specimen showed multiple yellowish nodules with septations (Figure 3A). On microscopic examination, nodular growth patterns with dense collagen bands were noticed (Figure 3B). Variable counts of lacunar-type Hodgkin/Reed–Sternberg (HRS) cells were identified in the background of mixed inflammatory cells (Figure 3C). Activated pneumocytes at the periphery of the lesion and multifocal aggregations of histiocytes were observed together (Figure 3D). In immunohistochemistry (IHC), tumor cells were CD30 and PAX5 positive. CD15 was positive in 5 cases. CD3 and CD20 were negative; however, one showed positivity of tumor cells for the T-cell marker, CD4.

### 3.3. Follow-Up and Survival Analysis

Bone marrow examination and positron emission tomography (PET) showed no involvement of other distant sites. Nine patients underwent additional chemotherapy (ABVD; Adriamycin, Bleomycin, Vinblastine, and Dacarbazine, #2 to #6), and all except one patient experienced complete remission. The patient who did not receive chemotherapy rapidly aggravated and died within one month.

### 3.4. Literature Review: Clinicopathological Features of 115 Cases

In the largest study of PPHL by Radin [5], he collected a total of 61 cases and analyzed the clinicopathological characteristics. After his study, there have been 44 cases reported from 1990 to 2021 [4,6,7,8,9,10,11,12,13,14,15,16,17,18,19,20,21,22,23,24,25,26,27,28,29,30,31,32,33,34,35,36,37,38]. Thus, a total of 115 cases including the present 10 cases were reviewed.

**Figure 4:**
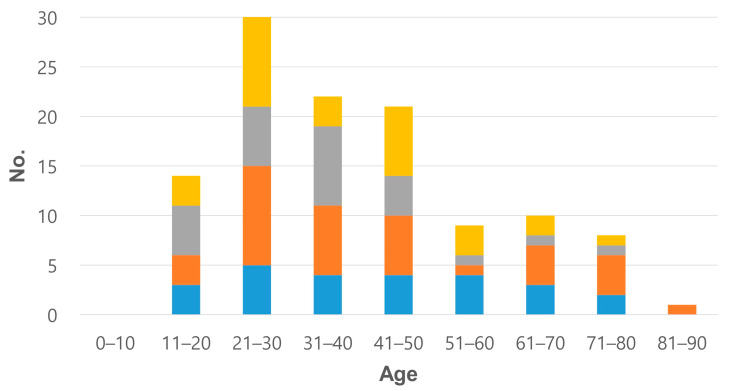
Age and sex distribution before and after 1990 (M, male; F, female).

**Figure 5:**
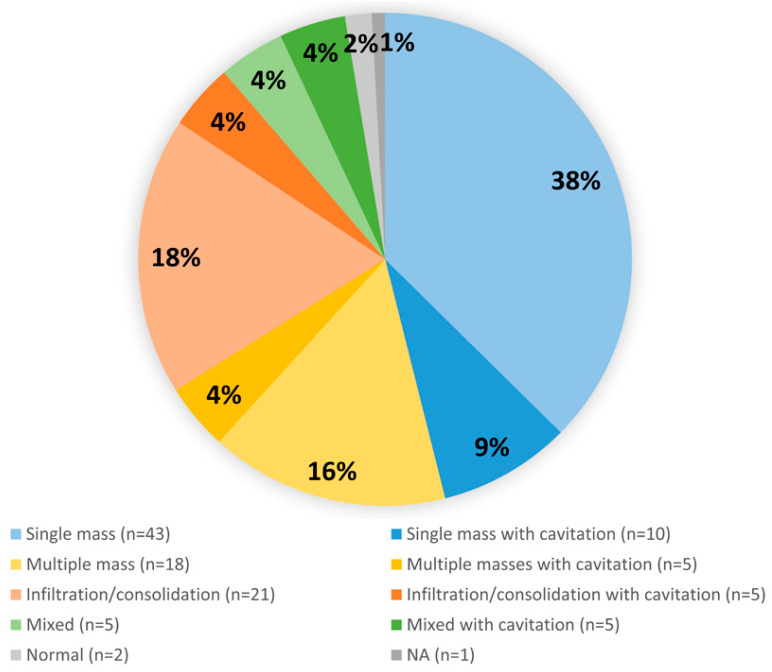
Analysis of radiological findings.

Female was affected more than male (64 of 115 cases, 1.25:1) and age distribution showed higher in the twenties (Figure 4; Supplementary Table S3). The most common subtype was nodular sclerosis (43/115, 37.4%) and mixed cellularity was the second common (20/115, 17.4%) (Table 2). In all radiological examinations, a single mass was the most common finding and some cases had multiple masses or infiltration/consolidation (Figure 5). Regional lymph node involvement was found in 39 of 100 cases (15 cases were not reported in the literature). The most common clinical impression was infectious pneumonia, including tuberculosis or abscess (55%, 22 of accessible 40 cases). In the initial needle biopsy of 61 cases, only 27 cases (44.3%) were diagnosed with ‘HL’ or ‘suspicious for/suggestive of HL’. Other initial pathologic diagnoses included chronic granulomatous inflammation, interstitial lymphocytic pneumonitis, carcinoma, solitary fibrous tumor, and Langerhans cell histiocytosis (Supplementary Table S4).

### 3.5. Differences in Clinicopathological Characteristics between Our 10 and Previously Published 105 Cases of PPHL

We compared the clinicopathological characteristics between ours (n = 10) and previous cases (n = 105), and significant differences were observed with respect to initial clinical impression and confirmatory method (Supplementary Table S5). Compared with previous cases, more of our cases were significantly suspected of being malignant at initial presentation (80% vs. 20%, *p* = 0.002) and confirmed pathologically at small biopsy or aspiration specimen (70% vs. 27.6%, *p* = 0.011). Many of our cases were initially diagnosed with a lymphoma but did not reach a statistical significance compared with previous cases (70% vs. 43.1%, *p* = 0.170). There were no significant differences in sex, age, CT finding, or HL subtype between ours and previous cases.

## 4. Discussion

Although pulmonary involvement of secondary HL is not uncommon and is reported to occur in 15–40% of patients, PPHL is extremely rare, and only about 100 cases have been reported since 1927 [25]. We collected 10 PPHL cases from two institutes. Sputum cytology, bronchial washing, and BAL fluid were mostly negative. Aspiration cytology can easily be under- or misdiagnosed due to inadequacy of specimen amount [7]. Exceptionally, one patient in this study had a Reed–Sternberg (RS)-like cell in aspiration cytology. Other than that, many PPHL can be recognized by lung biopsy and resection. Microscopically, mononuclear Hodgkin and multinucleated Reed–Sternberg cells are recognized in the background of mixed inflammatory infiltrates. Large and round nuclei with prominent nuclear membranes, pale chromatin, and eosinophilic nucleolus are identified in large cells with abundant, slightly basophilic cytoplasm. IHC can aim for the confirmative diagnosis of PPHL [18]. Tumor cells are usually positive for CD15, CD30, and PAX5 [2,18]. In immunohistochemical staining of 10 presented cases, all available cases are positive for CD30 and PAX5. CD3 and CD20 are negative but one case shows CD4 positivity, which is rare in HL [39] and can be a possible association with poor prognosis [40]. In this study, the patient with CD4 positivity had a complete remission.

However, it is still troublesome for pathologists when they encounter this challenging disease in limited samples [41]. It is difficult to recognize Hodgkin cells or Reed–Sternberg cells in small biopsies and on intraoperative frozen sections. Background infiltrates of inflammatory cells can be thought as nonspecific inflammation or hypersensitivity pneumonitis with increased eosinophils. Drug-associated lung disease is another pitfall that can also be misdiagnosed without proper clinical information. In addition, central necrosis with variable extent of histiocytes can be overlapped with histologic findings of tuberculosis [23]. Two patients in our study had anti-TB medication before surgical resection.

Beyond other pneumonia with necrosis or granulomatous inflammation, mesenchymal pulmonary tumor, such as inflammatory myofibroblastic tumor (IMT) is mentioned as another mimicker in the literature [41]. IMT has three histologic patterns (myxoid vascular pattern, compact spindle cell pattern, and hypocellular fibrous pattern) with many mixed inflammatory cells. ALK immunostaining might help exclude IMT. IgG4-related lung disease has also inflammatory background and compared with other organs, has fewer plasma cells and infrequent fibrosis [42]. Elevation of serum IgG4 level and accompanying autoimmune pancreatitis suggest the possibility of IgG4-related lung disease rather than PPHL [43]. So, in the inflammatory background, meticulous searching for Hodgkin and Reed–Sternberg (HRS) cells can reduce misdiagnosis. Another important differential diagnosis includes other epithelial or hematologic malignancies. The background of PPHL can be composed of diffuse and mixed-type inflammatory cells with entrapped pneumocytes having reactive atypia, so sufficient sample acquisition is necessary. In Langerhans cell histiocytosis, T-cell/histiocytic rich large B-cell lymphoma, peripheral T-cell lymphoma, anaplastic large cell lymphoma, and histiocytic sarcoma, tumor cells can be seen similarly to HRS cells. For excluding these hematologic malignancies, IHC and EBV-ISH are needed. Histologic characteristics and IHC tools for differential diagnosis are summarized in Table 3.

Epidemiologically, the largest past study of PPHL in 1990 had announced female predominance (1.4:1, F:M) and bimodal distribution of age (<35 and >60 years) [5]. Meanwhile, in a recent report by Cooksley et al. in 2014, the incidence is higher in the twenties [25], which is slightly different from our original 10 cases of two institutes but compatible with findings from an integrated literature review of 115 cases. Interestingly, before 1990, the bimodal age distribution is detected but the analysis of the literature review including all reports after 1990 highlights a single peak of young age (20–50 years). A possible hypothesis of this epidemiologic phenomenon includes that some pathophysiologic factors have provoked the earlier disease onset, recently. Advances in radiologic imaging or early screening test can be another explanation.

Patients can be either asymptomatic or manifest generalized and localized symptoms, such as weight loss, fever, night sweat, cough, sputum, and chest discomfort [5]. Radiologically, most tumors involve the upper portion of the lung presenting as solitary mass, alveolar consolidation, cavitation, or multiple nodules [5]. Nine of our 10 cases were also affected in upper lobe. In the overall review, single mass is the most common radiologic finding in PPHL (53 of 115, 46.1%). 18.9% (10/53) of single mass-presented cases have cavitation. Cases with multiple masses and pulmonary infiltration/consolidation have 21.7% (5/23) and 19.2% (5/26) accompanied by cavitary change, each. Except for mixed cases (5/10, 50%), about one-fifth is combined with cavitation. Pulmonary cavitary lesions on imaging should be considered with a wide differential diagnosis including granulomatous disease such as Wegener’s granulomatosis, bacterial or fungal infection, abscess, and metastasis [6].

Gross findings of PPHL are not commonly described because of the limited number of the resected specimen. However, in previous literature, PPHL showed a grayish-white friable mass and ill-defined infiltrative margins with necrosis and hemorrhage [11,17]. On gross examinations of our four resected specimens, multinodular whitish to yellowish masses were observed. Nevertheless, there are no definite pathognomonic radiologic/gross findings, so histological diagnosis with proper suspicion is still important.

Since treatment guidelines for PPHL have not been established, management is variable throughout the literature. If an initial biopsy is not adequate for the diagnosis of PPHL, surgical resection is required to exclude other diseases and confirm the diagnosis. Additionally, chemotherapy, as seen in this study, can be an effective treatment. There is no definite consensus about the chemotherapy regimen, but ABVD (Adriamycin, Bleomycin, Vinblastine, and Dacarbazine) is preferred in HL [28]. Even though radiation therapy was not performed for any of our 10 cases, a combination of chemotherapy and radiation after surgical resection appeared to be effective in reducing the recurrence rate [26].

Poor prognosis of PPHL has been reported to be associated with older age, B symptoms, bilaterality, multiplicity, pleural effusion, and cavitation [5,9,41,44]. Radin presented that PPHL was less favorable than nodal HL (13.2%, 5 of 38 progressed during therapy, and 47.4%, 18 of 38 relapsed after complete response) [5]. On the contrary, Nakachi et al. reported that 60.9% (14 of 23) survived and 17.4% (4 of 23) relapsed or died [45]. Of 10 presented cases in this study, the majority had complete remission. Only one patient died 1 month after diagnosis of PPHL. It suggests PPHL can be not as poor as previously reported and this improvement might be explained by the development of chemotherapy [18].

## 5. Conclusions

Clinicopathological experience for 10 cases of PPHL is meaningful as regards its rarity. Some cases initially went through misdiagnosis or underdiagnosis, but repeated biopsies or surgical procedures eventually confirmed the diagnosis. Especially, if a biopsy is not satisfactory, PPHL can mimic infectious or inflammatory disease (tuberculosis, granulomatous inflammation, etc.) as well as other pulmonary malignancies (adenocarcinoma). After the literature review, we found that younger patients are more affected by PPHL and variable radiologic findings including cavitation are observed. Treatment of PPHL has recently been improved in combination with chemotherapy (ABVD regimen) and has a better prognosis than previous studies. All these clinical, radiological, and pathological findings of PPHL may help pathologists as well as radiologists and clinicians to diagnose and manage this disease.

## Figures and Tables

**Table 1:** Clinical characteristics of our 10 PPHL cases.

Characteristic		Number of Cases
Age (years; range)		41 (27–72)
Sex	Man	4
	Woman	6
Smoking history	Never-smoker	7
	Ex-smoker	1
	Current smoker	2
Symptom	None	3
	Cough	6
	Chest pain	3
	Weight loss	1
	Sputum	1
CT findings	Single mass	8
	Consolidation	2
Mediastinal lymphadenopathy	Present	9
	Absent	1
Tumor location	Left upper lobe	5
	Left lower lobe	1
	Right upper lobe	4
Tumor size (cm; range)		5.7 (4.1–10.5)
Radiological impression	Carcinoma	7
	Sarcoma	1
	Mycobacterium	1
	Pneumonia	1
Treatment	Surgery followed by chemotherapy	4
	Chemotherapy	5
	Not performed	1
Treatment response	Complete remission	8
	Not applicable	2
Survival status (mean follow-up period)	Alive	9 (6.4 years)
	Dead	1 (1 month)
Sputum cytology	Not performed	8
	Negative	2
Bronchial washing cytology	Not performed	3
	Negative	7
Lung aspiration cytology	Not performed	6
	Negative	2
	Atypical cells	1
	Suspicious for Hodgkin lymphoma	1
Needle biopsy	Not performed	1
	Chronic granulomatous inflammation, suspicious for tuberculosis	1
	Atypical pneumocytes, suspicious for adenocarcinoma	1
	Hodgkin lymphoma	7
Subtype	Nodular sclerosis classical Hodgkin lymphoma	4
	Mixed cellularity classical Hodgkin lymphoma	2
	Lymphocyte-rich classical Hodgkin lymphoma	1
	Classic Hodgkin lymphoma unspecified	2
	Hodgkin lymphoma, not otherwise specified	1

**Table 2:** Distribution of subtypes.

Subtype	Number of Cases
Nodular sclerosis classical Hodgkin lymphoma	43
Mixed cellularity classical Hodgkin lymphoma	20
Lymphocyte-rich classical Hodgkin lymphoma	2
Nodular lymphocyte predominant Hodgkin lymphoma	1
Not applicable	49
Total	115

**Table 3:** Differential diagnosis of PPHL.

Category	Disease	Histologic Characteristics	IHC
Epithelial malignancy	Adenocarcinoma	Non-small cell carcinoma with glandular differentiation and variable architectural patterns (lepidic, acinar, papillary, micropapillary, and solid)	TTF1+, CK+
Histiocytic and dendritic cell neoplasm	Histiocytic sarcoma	Malignant proliferation of mature, large histiocytes showing abundant cytoplasm and often hemophagocytosis	CD68+, CD163+, lysozyme+
	Langerhans cell histiocytosis	Aggregates of Langerhans cells with convoluted and grooved nuclei in the background of eosinophils	S100+, CD1a+
B-cell neoplasm	T-cell/histiocyte-rich large B-cell lymphoma	Scattered large B-cells in the background of many T-cells and histiocytes forming diffuse or vaguely nodular patten	CD19+, CD20+, CD79a+, IgD–, BCL6–
	Lymphomatoid granulomatosis	Polymorphous lymphoid infiltrates with angiocentric distribution and often central necrosis	EBV+, CD20+, CD15–
T-cell neoplasm	PTCL	Diffuse infiltrates of medium to large neoplastic T-cells and inflammatory background	CD4±, CD8±, PAX5–
	ALCL	Large hallmark cells with horseshoe- or kidney-shaped nuclei	CD30+, ALK±
Mesenchymal tumor	IMT	Spindle shaped tumor cells with prominent inflammatory stroma	SMA+, ALK±
	Solitary fibrous tumor	Fibroblastic tumor cells with patternless architecture and staghorn vessels	STAT6 (+) CD34 (+)
Infection	Tuberculosis	Central necrosis surrounded by epithelioid histiocytes	
Inflammatory lesion	Hypersensitivity pneumonitis	Airway-centered inflammatory reaction in the clinical setting of exposure by causative agents	
	Bronchocentric granulomatosis	Necrotizing granulomatous inflammation involving bronchi and bronchioles	
	Interstitial lymphocytic pneumonitis	Diffuse interstitial infiltrates of lymphoplasma cells with lymphoid follicles and histiocytes	CD4+, CD8+
	Drug-associated lung disease	Variable degree of interstitial pneumonia by drug	
	IgG4-related disease	Lymphoplasmacytic infiltrates with storiform fibrosis, and obliterative phlebitis	CD138+, IgG4+

ALCL: anaplastic large cell lymphoma; ALK: anaplastic lymphoma receptor tyrosine kinase; CK: cytokeratin; IHC: immunohistochemistry; IMT: inflammatory myofibroblastic tumor; PTCL: peripheral T-cell lymphoma; TTF1: transcription termination factor 1; SMA: smooth muscle actin; STAT6: signal transducer and activator of transcription 6.

## Data Availability

Not applicable.

## Supplementary Materials

The following supporting information can be downloaded at: https://www.mdpi.com/article/10.3390/jcm12010126/s1, Table S1: Detailed clinicopathological characteristics of our 10 PPHL cases. Table S2: Immunostaining and EBER-ISH results of our 10 PPHL cases. Table S3: Demographic features of 115 PPHL cases. Table S4: Clinicopathologic features of 115 PPHL cases. Table S5: Differences in clinicopathological characteristics between our 10 cases and previously reported 105 cases of PPHL.

## References

[1] Hodgkin D. (1832). Absorbent glands and spleen: On some morbid appearances of the absorbent glands and spleen. Boston Med. Surg. J..

[2] Swerdlow S.H., Campo E., Harris N.L., Jaffe E.S., Pileri S.A., Stein H., Thiele J., Vardiman J.W. (2008). WHO Classification of Tumours of Haematopoietic and Lymphoid Tissues.

[3] Goldblum J.R., Lamps L.W., McKenney J.K., Myers J.L. (2017). Rosai and Ackerman’s Surgical Pathology.

[4] Van der Schee A.C., Dinkla B.A., van Knapen A. (1990). Primary pulmonary manifestation of Hodgkin’s disease. Respiration.

[5] Radin A.I. (1990). Primary pulmonary Hodgkin’s disease. Cancer.

[6] Lluch-Garcia R., Briones-Gomez A., Castellano E.M., Sanchez-Toril F., Lopez A., Brotons B. (2010). Primary pulmonary Hodgkin’s lymphoma. Can. Respir. J..

[7] Kumar R., Sidhu H., Mistry R., Shet T. (2008). Primary pulmonary Hodgkin’s lymphoma: A rare pitfall in transthoracic fine needle aspiration cytology. Diagn. Cytopathol..

[8] McElnay P.J., Pawade J., Chandratreya L., West D. (2013). Giant thoracic mass: An unusual presentation of primary pulmonary Hodgkin’s lymphoma. BMJ Case Rep..

[9] Chetty R., Slavin J.L., O’Leary J.J., Ansari N.A., Gatter K.C. (1995). Primary Hodgkin’s disease of the lung. Pathology.

[10] Boshnakova T., Michailova V., Koss M., Georgiev C., Todorov T., Sarbinova M. (2000). Primary pulmonary Hodgkin’s disease: Report of two cases. Respir. Med..

[11] Saad R.S., Leon M.E., Olson P.R. (2003). Pathologic Quiz Case: A localized pulmonary consolidation in a young woman. Arch. Pathol. Lab. Med..

[12] Codrich D., Monai M., Pelizzo G., Bussani R., Rabusin M., Guastalla P., Barbi E., Schleef J. (2006). Primary pulmonary Hodgkin’s disease and tuberculosis in an 11-year-old boy: Case report and review of the literature. Pediatr. Pulmonol..

[13] Pai R.R., Raghuveer C.V., Philipose R.T., Shetty A.B. (2006). Primary pulmonary Hodgkin’s disease: A distinct entity. Ind. J. Chest Dis. Allied Sci..

[14] Rodriguez J., Tirabosco R., Pizzolitto S., Rocco M., Falconieri G. (2006). Hodgkin lymphoma presenting with exclusive or preponderant pulmonary involvement: A clinicopathologic study of 5 new cases. Ann. Diagn. Pathol..

[15] Bakan N.D., Camsari G., Gur A., Ozkan G., Bayram M., Gorgulu F., Urer N. (2007). A 21-year-old male with productive cough, hemoptysis, chest pain, and weight loss. Respiration.

[16] Tillawi I.S. (2007). Primary pulmonary Hodgkin’s lymphoma. A report of 2 cases and review of the literature. Saudi Med. J..

[17] Malur P.R., Gaude G.S., Bannur H.B., Anurshetru S.B., Suranagi V.V., Kangle R.P., Dhumale A.J., Patil P.H., Davanagere R. (2009). Primary endobronchial Hodgkin’s disease. Lung India.

[18] Homma M., Yamochi-Onizuka T., Shiozawa E., Takimoto M., Ariizumi H., Nakashima H., Matsuda I., Nakamaki T., Kunimura T., Kushima M. (2010). Primary pulmonary classical Hodgkin lymphoma with two recurrences in the mediastinum: A case report. J. Clin. Exp. Hematop..

[19] Oka K., Shinonaga M., Nagayama R., Kashimura H., Yonekawa N., Tatebe S., Kuraoka S., Yatabe Y., Mori N. (2010). Coexistence of primary pulmonary Hodgkin lymphoma and gastric MALT lymphoma associated with Epstein-Barr virus infection: A case report. Pathol. Int..

[20] Binesh F., Halvani H., Taghipour S., Navabii H. (2011). Primary pulmonary classic Hodgkin’s lymphoma. BMJ Case Rep..

[21] Ezzine-Baccari S., Bacha D., Bouzaidi K., Ghrairi H., Sassi S. (2012). Hodgkin lymphoma presenting with exclusive pulmonary involvement. Tunis. Med..

[22] Simon Z., Jóna Á., Miltényi Z., Páyer E., Lieber A., Szilasi M., Illés Á. (2012). Diagnostic difficulties caused by a pulmonary infiltrate. Orv. Hetil..

[23] Valizadeh N., Gholamnejad M. (2012). Pulmonary Hodgkin lymphoma misdiagnosed as tuberculosis. Int. J. Hematol. Oncol. Stem. Cell Res..

[24] Fratoni S., Abruzzese E., Niscola P., Trawinska M.M., Mercadante E., Casullo A., de Fabritiis P., Perrotti A., Santeusanio G. (2013). Primary pulmonary Hodgkin lymphoma simulating a mediastinal tumour: An uncommon occurrence. Mediterr. J. Hematol. Infect. Dis..

[25] Cooksley N., Judge D.J., Brown J. (2014). Primary pulmonary Hodgkin’s lymphoma and a review of the literature since 2006. BMJ Case Rep..

[26] Schild M.H., Wong W.W., Valdez R., Leis J.F. (2014). Primary pulmonary classical Hodgkin lymphoma: A case report. J. Surg. Oncol..

[27] Tanveer S., El Damati A., El Baz A., Alsayyah A., ElSharkawy T., Regal M. (2015). Primary pulmonary Hodgkin lymphoma. Rare Tumors.

[28] El Hage H., Hossri S., Samra B., El-Sayegh D. (2017). Primary pulmonary Hodgkin’s lymphoma: A rare etiology of a cavitary lung mass. Cureus.

[29] Lowenthal B.M., Xu X., Subash M., Jih L.J. (2017). Hodgkin’s lymphoma with unusual pulmonary presentations: Reporting two cases. Ind. J. Pathol. Microbiol..

[30] Abid H., Khan J., Lone N. (2018). Hodgkin’s Lymphoma presenting as an obstructing endobronchial mass: A rare presentation. BMJ Case Rep..

[31] Aljehani Y., Al-Saif H., Al-Osail A., Al-Osail E. (2018). Multiloculated cavitary primary pulmonary hodgkin lymphoma: Case series. Case Rep. Oncol..

[32] Conti L., Pisani D., Gatt A., Montefort S. (2018). Unusual case of primary pulmonary Hodgkin’s lymphoma presenting with a continuous murmur. BMJ Case Rep..

[33] Parente P., Zanelli M., Zizzo M., Carosi I., Di Candia L., Sperandeo M., Lacedonia D., Fesce V.F., Ascani S., Graziano P. (2020). Primary pulmonary Hodgkin lymphoma presenting as multiple cystic lung lesions: Diagnostic usefulness of cell block. Cytopathology.

[34] Chowdhary G.S., Mehta R., Tyagi R. (2020). Primary pulmonary Hodgkin’s lymphoma with pulmonary histoplasmosis. Med. J. Armed Forces India.

[35] Bertoglio P., Lomangino I., Querzoli G., Bonalumi A., Bogina G.S., Terzi A.C. (2021). Primary Hodgkin lymphoma of the lung arising with hemoptysis and pulmonary consolidation: A case report. Monaldi Arch. Chest Dis..

[36] Chiu W.C., Chen S.H., Chen B.J., Huang Y.L., Miserc J.S., Wei C.H., Lin W.C. (2021). Primary pulmonary Hodgkin’s lymphoma: A rare etiology mimicking pulmonary tuberculosis. Pediatr. Neonatol..

[37] Kanitra J.J., Thampy C.A., Cullen M.L. (2021). A decade’s experience of pediatric lung abscess and empyema at a community hospital. Pediatr. Pulmonol..

[38] Sun K., Yu Q., Zhou J., Zhang H., Gao L., Nong L., Wang M., Que C. (2021). Primary pulmonary Hodgkin’s lymphoma mimicking rheumatoid arthritis-associated organizing pneumonia: A case report. Thorac. Cancer.

[39] Tzankov A., Bourgau C., Kaiser A., Zimpfer A., Maurer R., Pileri S.A., Went P., Dirnhofer S. (2005). Rare expression of T-cell markers in classical Hodgkin’s lymphoma. Mod. Pathol..

[40] Venkataraman G., Song J.Y., Tzankov A., Dirnhofer S., Heinze G., Kohl M., Traverse-Glehen A., Eberle F.C., Hanson J.C., Raffeld M.A. (2013). Aberrant T-cell antigen expression in classical Hodgkin lymphoma is associated with decreased event-free survival and overall survival. Blood.

[41] William J., Variakojis D., Yeldandi A., Raparia K. (2013). Lymphoproliferative neoplasms of the lung: A review. Arch. Pathol. Lab. Med..

[42] Patel M., Kumar B., Diep M.L., Nandurkar D. (2016). IgG4 related lung disease. Can. Respir. J..

[43] Hirano K., Kawabe T., Komatsu Y., Matsubara S., Togawa O., Arizumi T., Yamamoto N., Nakai Y., Sasahira N., Tsujino T. (2006). High-rate pulmonary involvement in autoimmune pancreatitis. Int. Med. J..

[44] Yousem S.A., Weiss L.M., Colby T.V. (1986). Primary pulmonary Hodgkin’s disease. A clinicopathologic study of 15 cases. Cancer.

[45] Nakachi S., Nagasaki A., Owan I., Uchihara T., Fujita J., Ohshima K., Miyagi T., Taira T., Taira N., Takasu N. (2007). Primary pulmonary Hodgkin lymphoma: Two case reports and a review of the literature. Gan Kagaku Ryoho..

