# Supplementary material for: Clinicopathological Characteristics of Primary Pulmonary Hodgkin Lymphoma (PPHL): Two Institutional Experiences with Comprehensive Literature Review of 115 PPHL Cases
Source: J Clin Med. 2022 Dec 23;12(1):126. doi: 10.3390/jcm12010126 (PMC9821715; doi:10.3390/jcm12010126)
Supplement: Supplementary file 1 [file jcm-12-00126-s001.zip › jcm-2111586-supplementary.pdf]

Supplementary Table S1. Detailed clinicopathological characteristics of our 10 PPHL cases.

**Case No**	**1**	**2**	**3**	**4**	**5**	**6**	**7**	**8**	**9**	**10**
Sex	Female	Male	Male	Female	Male	Female	Female	Female	Female	Male
Age at diagnosis (years)	71	37	39	41	33	47	27	41	48	72
Smoking history	None	Current	Current	None	Ex	None	None	None	None	None
Symptom	Cough	Cough, sputum, chest discomfort	Cough, chest pain	Left axillary pain	Cough, weight loss	None	Cough	None	None	Cough
Radiological findings	Single mass, 4.5 cm	Single mass, 4.3 cm	Single mass, 6.5 cm	Single mass, 4.1 cm	Single mass, 5.0 cm	Single mass, 7.8 cm	Single mass, 10.5 cm	Single mass, 6.4 cm	Consolidation	Consolidation
Mediastinal LAP	Present	Present	Present	Present	Present	Present	Present	Present	Absent	Present
Sputum cytology	Negative	Negative	ND	ND	ND	ND	ND	ND	ND	ND
Bronchial washing cytology	Negative	Negative	Negative	ND	Negative	Negative	ND	ND	Negative	Negative
Lung aspiration cytology	Negative	A single RS-like cell	ND	ND	Negative	ND	Rare atypical cells	ND	ND	ND
Needle biopsy diagnosis (location)	Mixed cellularity HL (MLN)	Lymphocyte-rich HL (lung)	Suggestive of HL (lung)	Atypical pneumocytes (lung)	Classic HL, unspecified (lung)	CGI with necrosis (lung)	Nodular sclerosis HL (lung)	Classic HL, unspecified (lung)	ND	Mixed cellularity HL (lung)
Preoperative management	None	None	None	NAC	None	Anti-TB medication	None	None	Anti-TB medication	Antibiotics
Surgical resection (diagnosis)	ND	Lymphocyte-rich HL	ND	Nodular sclerosis HL	ND	Nodular sclerosis HL	ND	ND	Nodular sclerosis HL	ND
Treatment	CTx	CTx	CTx	CTx	CTx	CTx	CTx	CTx	CTx	ND
Response	NA (LTF)	CR	CR	CR	CR	CR	CR	CR	CR	NA
Survival status	Alive	Alive	Alive	Alive	Alive	Alive	Alive	Alive	Alive	Dead
Observation period	4 years	21 years	8 years	5 years	2 years	5 years	5 years	4 years	4 years	1 month

CGI: chronic granulomatous inflammation; CTx: Chemotherapy; CR: complete remission; HL: Hodgkin lymphoma; LAP: lymphadenopathy; LTF: lost to follow-up; MLN: mediastinal lymph node; NAC: neoadjuvant chemotherapy; ND: not done; PD: progressive disease; PR: partial response; RS: Reed-Sternberg; TB: tuberculosis.

Supplementary Table S2. Immunostaining and EBER-ISH results of our 10 PPHL cases.

**Case No**	**1**	**2**	**3**	**4**	**5**	**6**	**7**	**8**	**9**	**10**
Histological subtype	Mixed cellularity	Lymphocyte -rich	NOS	Nodular sclerosis	Unspecified	Nodular sclerosis	Nodular sclerosis	Unspecified	Nodular sclerosis	Mixed cellularity
CD30	Positive	ND	Positive	Positive	Positive	Positive	Positive	Positive	Positive	Positive
CD15	ND	Positive	Negative	Focal positive	Negative	Focal positive	Positive	Equivocal	Positive	Equivocal
CD20	ND	Negative	Negative	ND	Negative	Negative	Negative	Negative	Negative	Negative
PAX5	ND	ND	Positive	Positive	Positive	Positive	Positive	Positive	Positive	Positive
CD3	ND	Negative	Negative	ND	Negative	ND	ND	Negative	Negative	Negative
CD4	ND	ND	Negative	ND	ND	ND	ND	ND	Positive	ND
EBER-ISH	ND	ND	Negative	Negative	Negative	Negative	Negative	Negative	Negative	Negative

ND: not done; NOS: not otherwise specified.

Supplementary Table S3. Demographic features of 115 PPHL cases.

Age (years)	0–10	11–20	21–30	31–40	41–50	51–60	61–70	71–80	81–90	Total
Number of cases	0	14	30	22	21	9	10	8	1	115
Male (M)	0	8	11	12	8	5	4	3	0	51
Female (F)	0	6	19	10	13	4	6	5	1	64
M:F before 1990	0:0	3:3	5:10	4:7	4:6	4:1	3:4	2:4	0:1	25:36 (1:1.44)
M:F from 1990	0:0	5:3	6:9	8:3	4:7	1:3	1:2	1:1	0:0	26:28 (1:1.08)

Supplementary Table S4. Clinicopathologic features of 115 PPHL cases.

Author (year published)	Sex (number of cases)	Age	Radiology	LAP (number of cases)	Initial clinical impression (number of cases)	Initial pathological diagnosis (number of cases)	Confirmatory method (number of cases)	Histological subtype (number of cases)
Radin (1990)	F (36); M (25)	19-82 (F); 12-77 (M)	Single mass (17); single mass with cavitation (8); multiple mass (16); multiple mass with cavitation (1); infiltration/ consolidation (8); infiltration/consolidation with cavitation (3); mixed (4); mixed with cavitation (1)	Yes (15); no (31); NA (15)	Abscess or HL (1); carcinoma (1); bronchial adenoma (1); pneumonia (1); eosinophilic pneumonitis (1); NA (56)	HL or suggestive of HL (11); non-diagnostic (3); interstitial lymphocytic pneumonitis (1); NA (46)	Initial biopsy (11); repeated biopsy (1); surgical resection (21); postmortem (2); NA (26)	NS (16); MC (10); LP (1); NA (34)
Schee et al. (1990)	M	46	Mass	Yes	NA	Non-diagnostic	Segmental resection	NS
	F	31	Multiple nodules	No	NA	Non-diagnostic	Wedge resection	MC
Chetty et al. (1995)	F	24	Mass	No	NA	Atypical large cells	Open lung biopsy	NA
	M	48	Consolidation	Yes	NA	NA	Open lung biopsy	NA
	M	58	Mass	No	NA	NA	Open lung biopsy	NA
Boshnakova et al. (2000)	M	15	Mass	No	NA	Eosinophilic granuloma	Lobectomy	NS
	M	42	Reticular infiltrates	No	TB; pneumonia	Interstitial lymphocytic pneumonitis and fibrosis	Autopsy	MC
Rodriguez et al. (2006)	M	46	Mass	No	Neoplasm	NA	Pneumonectomy	MC
	M	17	Consolidation	No	Pneumonia	Malignant lymphoma; Langerhans cell histiocytosis	Wedge biopsy	NS
	M	34	Consolidation	No	NA	Bronchiocentric granulomatosis; lymphomatoid granulomatosis; large cell lymphoma	Lobectomy	MC
	F	42	Mass	Yes	Neoplasm	NA	Bronchial biopsy	MC
	F	48	Mass	Yes	Neoplasm	Poorly differentiated carcinoma	Pneumonectomy	MC
Codrich et al. (2006)	M	11	Parenchymal density	No	Atelectasis due to foreign body/mucus plug, atypical	Chronic inflammation	Lobectomy	MC

					pneumonia, or TB; infected pulmonary congenital malformation; malignant nodal enlargement			
Pai et al. (2006)	F	30	Solitary nodular lesion	No	Round pneumonia; TB	Non-diagnostic	Wedge biopsy	NS
Bakan et al. (2007)	M	21	Cavitary lesion and consolidation	Yes	Lung abscess; TB; complicated hydatid cyst; septic emboli; Wegener's granulomatosis	Pneumonia	Wedge biopsy	NA
Tillawi (2007)	M	21	Mass	No	Langerhans cell histiocytosis; Wegener's granulomatosis	Non-diagnostic	Wedge biopsy	NS
	F	32	Mass	Yes	Infectious condition	Non-diagnostic	Wedge biopsy	NS
Saad et al. (2003)	F	21	Consolidation	No	Pneumonia; pulmonary embolism	NA	Segmentectomy	NS
Kumar et al. (2008)	M	36	Mass	No	Infectious condition	Non-small cell lung carcinoma	Lymph node biopsy	NS
Malur et al. (2009)	F	43	Mass	No	NA	Inflammatory lesion	Resection	NA
Lluch-Garcia et al. (2010)	M	21	Cavitary lesion	Yes	Pulmonary abscess	Suspicious for HL	Wedge biopsy	NS
Homma et al. (2010)	F	58	Cavitary mass	No	NA	Suspected adenocarcinoma	Pneumonectomy	NS
Oka et al. (2010)	F	64	Mass	No	NA	NA	Resection	NS
Binesh et al. (2011)	F	54	Multiple nodules	No	NA	NA	Wedge biopsy	NS
Valizadeh et al. (2012)	M	28	Consolidation	No	TB	Chronic non-caseating granulomatous inflammation	Wedge biopsy	NA
Simon et al. (2012)	M	30	Consolidation	No	Atypical pneumonia; TB	Non-diagnostic	Resection	LR

Ezzine-Baccari et al. (2012)	F	23	Consolidation with cavity	No	NA	Granulation tissue, suspicious for TB	Wedge biopsy	MC
McElnay et al. (2013)	F	61	Mass	Yes	NA	Solitary fibrous tumor	Resection	NS
Fratoni et al. (2013)	F	27	Mass	No	NA	Non-diagnostic	Wedge biopsy	NS
Cooksley et al. (2014)	F	28	Consolidation/mass with multiple cavities	No	TB; congenital cystic adenoid malformation	Non-specific acute inflammation; Langerhans cell histiocytosis	Wedge biopsy	NS
Schild et al. (2014)	F	53	Mass	No	NA	Acute and organizing pneumonia	Lobectomy	NS
Tanveer et al. (2015)	F	15	Consolidation	No	TB	Non-diagnostic	Lobectomy	NS
Lowenthal et al. (2017)	M	35	Consolidation with cavitation and multiple cavitary nodules	No	TB	NA	Transbronchial biopsy	NA
El Hage et al. (2017)	F	27	Multiple mass with cavitation	Yes	Community-acquired pneumonia	Suspicion of HL	CT-guided biopsy	NS
Abid et al. (2018)	M	40	Cavitary consolidation	No	Bronchitis; lung cancer	Consistent with HL	Bronchoscopic biopsy	NA
Aljehani et al. (2018)	M	16	Multiloculated cavitary lesion	Yes	NA	Non-diagnostic	Wedge biopsy	NS
	F	29	Multiloculated cavitary mass	Yes	NA	Consistent with HL	CT-guided biopsy	NS
Conti et al. (2018)	F	33	Mass	Yes	Asthma; lymphoma	HL	CT-guided biopsy	NS
Parente et al. (2020)	F	18	Multiple cavitary lesions	No	NA	HL	Fine needle aspiration	NA
Chwedhary et al. (2020)	M	24	Mass	No	NA	HL with histoplasmosis	Open biopsy	NA
Bertoglio et al. (2021)	M	37	Consolidation	Yes	TB	Infection; granulomatosis with polyangiitis	Resection	Classic

Chiu et al. (2021)	M	16	Consolidation	Yes	TB	HL	Resection	NS
Kanitra et al. (2021)	F	15	Fluid collection and multiloculated effusion with cavitation	Yes	Necrotizing pneumonia	HL	CT-guided biopsy	NA
Sun et al. (2021)	M	61	Multiple consolidation and nodules	No	Seronegative rheumatoid arthritis-associated organizing pneumonia	Organizing pneumonia	VATS biopsy	NS
Jung et al. (2022; present study)	F	71	Mass	Yes	Lung cancer	HL	CT-guided biopsy	MC
	M	37	Mass	Yes	Lung cancer	HL	CT-guided biopsy	LR
	M	39	Mass	Yes	Lung cancer	Suggestive of HL	CT-guided biopsy	NA
	F	41	Mass	Yes	Lung cancer	Atypical pneumocytes	Resection	NS
	M	33	Mass	Yes	Lung cancer	HL	CT-guided biopsy	NA
	F	47	Mass	Yes	Lung cancer	CGI with necrosis	Resection	NS
	F	27	Mass	Yes	Pulmonary sarcoma	HL	CT-guided biopsy	NS
	F	41	Mass	Yes	Lung cancer	HL	CT-guided biopsy	NA
	F	48	Consolidation	No	TB	Non-diagnostic	Resection	NS
	M	72	Consolidation	Yes	Pneumonia	HL	CT-guided biopsy	MC

CGI: chronic granulomatous inflammation; CT: computed tomography; F: female; HL: Hodgkin lymphoma; LAP: lymphadenopathy; M: male; MC: mixed cellularity; NA: not applicable; NS: nodular sclerosis; LP: lymphocyte predominant; LR: lymphocyte-rich; TB: tuberculosis; VATS: video-assisted thoracoscopic surgery.

Supplementary Table S5. Differences in clinicopathological characteristics between our 10 cases and previously reported 105 cases of PPHL.

**Characteristic**		**Number of cases (%)**		**Fisher's exact *p*-value**
		Our cases (n=10)	Previous cases (n=105)	
Sex	Male	4 (40)	47 (44.7)	1.000
	Female	6 (60)	58 (55.2)	
Age (years)	≤40	4 (40)	62 (59.0)	0.320
	>40	6 (60)	43 (40.9)	
CT finding	Single mass	8 (80)	45 (43.2)	0.484
	Consolidation	2 (20)	24 (23.0)	
Initial clinical Impression	Malignancy	8 (80)	6 (20)	0.002*
	Inflammatory lesion	2 (20)	21 (70)	
Initial pathologic diagnosis	Lymphoma	7 (70)	22 (43.1)	0.170
	Others/Non-diagnostic	3 (30)	29 (56.8)	
Confirmatory method	Small biopsy/aspiration	7 (70)	21 (27.6)	0.011*
	Open biopsy/resection	3 (30)	55 (72.3)	
Subtype	Nodular sclerosis	4 (40.0)	39 (66.1)	0.115
	Others	6 (60.0)	20 (33.8)	

*Statistically significant.
